# Evaluation of the local decoupling of livestock and cropland in the Huang-Huai-Hai region

**DOI:** 10.1007/s11356-022-21993-2

**Published:** 2022-07-25

**Authors:** Zhiwei Du, Yuexian Liu, Jingtao Ding, Guoyuan Zou, Zhengyi Hu, Ruili Zhang

**Affiliations:** 1 College of Resources and Environment, University of Chinese Academy of Sciences, Beijing, 100049 China; 2 Ministry of Agriculture and Rural Affairs, Academy of Agricultural Planning and Engineering, Beijing, 100125 China; 3 Institute of Plant Nutrition and Resources, Beijing Academy of Agriculture and Forestry Science, Beijing, 100097 China

**Keywords:** Land carrying capacity, Animal manure absorption capacity, Risk warning value, Optimal options, Recoupling

## Abstract

Decoupling livestock and cropland production at regional scale have poor resource-use efficiency and detrimental effects on environment in China. It is therefore necessary to identify the decoupled livestock and cropland production system and make recommendations to recouple livestock and cropland. This study used the indexes of land carrying capacity (*LCC*), animal manure absorption capacity (*AMAC*), and risk warning value (*R*) to evaluate the coupling between cropland and livestock at the local scale in the Huang-Huai-Hai region. The decoupling of cropland and livestock in the case of Beijing (SY_BJ) was found assessed with lower theoretical value of *LCC* and higher theoretical value of *AMAC* compared with local actual situation, categorized as grade IV with a high *R* value (above 1). Contrary results were found that the livestock and cropland production systems were coupled at the local scale in the cases located in Hebei and Shandong Provinces, categorized as grade I or II. Two measures were used to optimize the decoupled case by adjusting the ratio of manure to fertilization or reducing breeding quantity. The decoupled case of SY_BJ could be optimized by adjusting the ratio of manure to fertilization (95.34% based on nitrogen and 81.97% based on phosphorus, respectively). The breeding quantity in this case should be reduced by at least 46% to recouple the livestock and cropland at the local level to manage nutrient surpluses from livestock and poultry breeding.

**Supplementary Information:**

The online version contains supplementary material available at 10.1007/s11356-022-21993-2.

## Introduction

Chinese farmers have a long history of crop fertilization with livestock, poultry, and human excrement (Hou et al. 2018; Wang et al. 2018). Driven by the increased demand for protein-based food, breeding livestock and poultry in small farm backyards has transitioned to industrial livestock farms, which are typically located far from croplands (Bai et al. 2018; Wang et al. 2018). The share of rural households with both crop planting and livestock raising has sharply declined from 71% in 1986 to only 12% in 2017 across China (Jin et al. 2021). This decoupling of livestock production and croplands has substantially reduced the amount of manure recycling and has had detrimental effects on the environment (Sun et al. 2012; Sutton et al. 2013; van Grinsven et al. 2018; Zhuang et al. 2019; Gu 2022). In North and Latin America, some large confined animal feeding operations seem to couple with extensive areas of cropland, but remain persistent hotspots of nitrogen loss (Franzluebbers et al. 2021). Non-agricultural-sector activities, such as mechanization, synthetic fertilizer use, and the income share, are also the main driving forces of decoupling in China, which have notable adverse effects on draft-animal raising and the proportion of crop planting and livestock raising (Qian et al. 2018; Zhang et al. 2019; Jin et al. 2021). Thus, rebuilding the linkage between livestock and cropland is a crucial step towards nutrient recycling and sustainable intensification (Jin et al. 2021), and is also a crucial pathway to reduce the environmental pollution risk of livestock manure (Chen et al. 2014; Zhang et al. 2019).

The recoupling of livestock and cropland has attracted attentions from various stakeholders (Ma et al. 2018; Saikia et al. 2019). Bai et al. (2022) demonstrate that relocating one-third of livestock in China to match the distribution of croplands would reduce manure nitrogen emissions by two-thirds and halve the number of people exposed to high NH_3_ emissions from manure. Ma et al. (2018) found that the recoupling of livestock and cropland through the exchange of feed for manure seems to be an economic and environmental win–win situation. Resource use of animal manure from the livestock sectors to farmland can supplement to chemical fertilizers (Wang et al. 2021a, b). Livestock relocation was explored from West European countries to Central European countries (Van Grinsven et al. 2015, 2018). Most of these studies focusing on the connection between livestock production and cropland are at the regional scale, since a regional approach could provide vital opportunities for the sustainable intensification of agriculture (Jin et al. 2021). However, cropland carrying capacity to absorb manure nitrogen is significant differences at the regional scale in China due to two main reasons (Zhang et al. 2019; Wang et al. 2021a, b). The first one is that nitrogen demand by crops was less than the amount of livestock excretion N in 74% of Chinese provinces. Another reason is that the centralized feedlots are far from farmland areas capable of absorbing manure nitrogen (Zhang et al. 2019). One potential way to reduce pollution risk is to export excess livestock manure from intensive peri-urban livestock farming regions to adjacent areas in which manure is scarce (Jia et al. 2015; Zhang et al. 2019; Wen et al. 2020). Therefore, it is urgent to determine the amount of livestock manure returning to local cropland area based on theoretical guidance.

In the discussion of the recoupling degree of livestock and cropland at local scale, the assessment index has received increasing attention. The index of pig/farmland ratio (PFR) was used to reflect the recoupling degree between livestock and croplands (Jin et al. 2021). The index of livestock manure N or P load on farmland has been proposed to estimate the carrying capacity of local livestock farms (Jia et al. 2015; Yang et al. 2016; Yan et al. 2017; Zhang et al. 2019) and identified the environmentally critical regions of livestock farming in mainland China (Zheng et al. 2019). Li and Liu (2020) used the soil’s own nutrient-supplying capacity as an empirical method to accurately determine the carrying capacity and environmental risks of the livestock and poultry breeding in Jiangsu Province. Wen et al. (2020) compared the farmland area required for the reasonable consumption and utilization of the livestock manure nitrogen nutrient with the actual farmland area to assess the risk in Wuhan. Zhu et al. (2020) calculated the nutrients of livestock manure to explore the land carrying capacity of livestock and poultry manure using the calculation method of the Ministry of Agriculture and Rural Affairs. Other indexes, such as environmental carrying capacity for livestock and poultry farming, have also been used to assess the environmental impact of the livestock and poultry farming industry (Peng and Bai 2013). However, the above studies mainly used only one index to assess the potential of returning livestock and poultry excrement as nutrient resources for crop growth. Less information is available on the comparative assessment of decoupling degree between livestock and cropland production at local scale. The multi-index, such as the land carrying capacity (*LCC*), animal manure absorption capacity (*AMAC*), and risk warning value (*R*), can be combined to assess the local coupling degree and compare decoupling at different counties considering their spatial variations.

The Huang-Huai-Hai (HHH) region is representative of intensive cultivation areas and has the highest amount of animal manure in China. In 2017, there were 288 million tons of livestock manure and 180 million tons of urine produced in the HHH region. Moreover, this area has high pollution emission load, with one-third of China’s nitrogen and phosphorus being emitted from this area, even though it only represents one-sixth of the land area (The Statistics Yearbook 2018). Recycling these nutrients has been a great challenge for the HHH region in the context of sustainable intensification of livestock and poultry breeding. The application of animal manure in the HHH region is highly context specific with substantial variation in the rate of manure return to the field. The effect of this variation on the local coupling and decoupling of the livestock and croplands is not yet well understood and requires further research. Therefore, we studied four counties in this region with the aim to determine (1) the extent to which local decoupling between livestock and cropland production occurs at the county scale, with the integrity of three indexes, such as the *LCC*, *AMAC*, and risk warning (*R*); (2) the main driving forces to the decoupling at the local scale; and (3) the optimal options for future recoupling between livestock and croplands in the identified decoupled case.

## Materials and methods

### Study areas

**Fig. 1:**
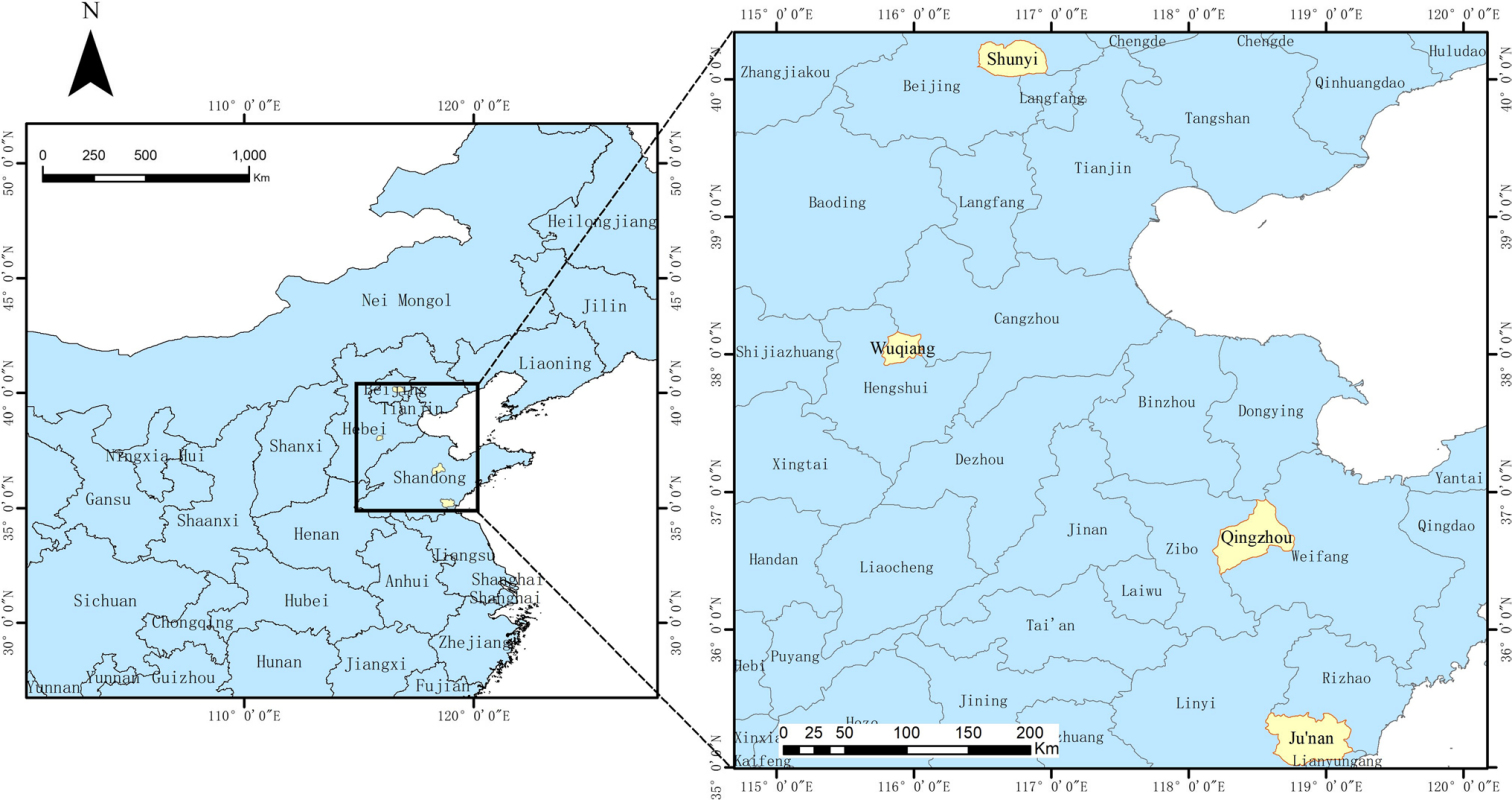
Locations of the study sites in the HHH region

The study areas are located in the HHH region (Fig. 1). The main body of the HHH region is alluvial plain formed by the downstream region of the Yellow River basin, the Huaihe River basin, and the Haihe River basin, which covers an area of approximately 300,000 m^2^ (Lu et al. 2013). This area not only has a long history of farming, but is also an intensive breeding area, accounting for 20–40% of the total meat, egg, and milk production in China. Four counties were identified in this region characterized by intensive livestock and poultry farming to reflect differences in the coupling or decoupling the livestock and cropland, and abbreviated according to county and province as follows: SY for Shunyi County, QZ for Qingzhou County-level City, JN for Junan County, WQ for Wuqiang County, BJ for Beijing, SD for Shandong Province, and HB for Hebei Province. The scope of the study covered livestock, poultry, and cropland throughout the region.

### Data sources

Our analysis applied three main data sources. Firstly, the Technical Guide, including data on recommended amount of nitrogen and phosphorus required by 100 kg production of crops, and nutrient proportion of fertilizer under different nitrogen and phosphorus nutrient levels in soil (Tables 1 and 2). Secondly, the China Pollution Sources Census (CPSC), including parameters and pollution coefficients (Table 3). Finally, the Statistical Yearbook of Beijing Shunyi, Shandong Linyi and Weifang, and Hebei Hengshui, including data on crop yield planting area, and livestock inventory. A detailed description of the data sources is given in Table S1 and  S2.

**Table 1:** Nitrogen and phosphorus demand per 100 kg outputs of crop (*i*)

Crop species		Nitrogen kg·100^−1^·kg^−1^ (*Q*_*i*_)	Phosphorus kg·100^−1^·kg^−1^ (*Q*_*i*_)
Staple crops	Wheat	3.00	1.00
	Rice	2.20	0.80
	Corn	2.30	0.30
	Millet	3.80	0.44
	Soybeans	7.20	0.75
	Cotton	11.70	3.04
	Potato	0.50	0.09
Vegetables	Cucumber	0.28	0.09
	Tomato	0.33	0.1
	Green pepper	0.51	0.11
	Eggplant	0.34	0.10
	Chinese cabbage	0.15	0.07
	Radish	0.28	0.06
	Green Chinese onion	0.19	0.04
	Garlic	0.82	0.15
Fruits	Peach	0.21	0.03
	Grape	0.74	0.51
	Banana	0.73	0.22
	Apple	0.30	0.08
	Pear	0.47	0.23
	Citrus	0.60	0.11
Economic crops	Oil plants	7.19	0.89
	Sugarcane	0.18	0.02
	Beet	0.48	0.06
	Tobacco	3.85	0.53
	Tea	6.40	0.88

MOA (2018)

**Table 2:** Nutrient proportion of fertilizer under different nitrogen and phosphorus nutrient levels in soil

Classification of soil nitrogen and phosphorus		I	II	III
Proportion of fertilizer nutrients in the total nutrient demand (*FP*)		35%	45%	55%
Total nitrogen content in soil (g·kg^−1^)	Dry land	> 1.0	0.8–1.0	< 0.8
	Paddy field	> 1.2	1.0–1.2	< 1.0
	Vegetable field	> 1.2	1.0–1.2	< 1.0
	Orchard	> 1.0	0.8–1.0	< 0.8
Available phosphorus content in soil (mg·kg^−1^)		> 40	20–40	< 20

MOA (2018)

**Table 3:** Excretion coefficient of livestock and poultry breeding

Area	Animal species	Feeding stage	Pollutant index		Unit	Excretion coefficient (*X*_*k*_)
North China	Pig	Conservation	Fecal		kg·pig^−1^·day^−1^	1.04
			Urine		L·pig^−1^·day^−1^	1.23
				TN	g·pig^−1^·day^−1^	20.40
				TP	g·pig^−1^·day^−1^	3.48
		Fattening	Fecal		kg·pig^−1^·day^−1^	1.81
			Urine		L·pig^−1^·day^−1^	2.14
				TN	g·pig^−1^·day^−1^	33.23
				TP	g·pig^−1^·day^−1^	6.06
		Pregnant	Fecal		kg·pig^−1^·day^−1^	2.04
			Urine		L·pig^−1^·day^−1^	3.58
				TN	g·pig^−1^·day^−1^	43.66
				TP	g·pig^−1^·day^−1^	9.93

CPSC: Handbook of Pollution Excretion Coefficients of the Livestock and Poultry Industry

### Estimation of *LCC* and *AMCC*

The *LCC* refers to the maximum amount of livestock that can be carried by the region if all the manure production is applied to crop planting (Zheng et al. 2019). The calculation method was taken from the Technical Guide (MOA 2018):

1 $$LCC=\frac{\Sigma \left(Y\times {Q}_{i}\right)\times FP\times MP}{MR\times {X}_{k}\times RR}$$

where $LCC$ is the land carrying capacity; *Y* is total yield of each kind of crop (Table S1); *Q*_*i*_ is the N/P demand per 100 kg outputs of crop *i* (Table 1); *FP* is the proportion of fertilizer nutrients in the total nutrient demand (Tables 2 and 4); *MP* is the ratio of manure nutrients to total quantity of fertilizer nutrients (*MP* of N/P is 50%, Table 4); *MR* is seasonal utilization rate of manure (*MR* of N is 25–35%, and *MR* of P is 30–35%, Table 4); *X*_*k*_ is the excretion coefficient of pig equivalent (Table 3); and *RR* is the nutrient retention rate (*RR* of N is 63.5%, *RR* of P is 68.5%, Table 4).

**Table 4:** The coefficient for calculation of *LCC* and *AMAC*

Coefficient item		Value	Reference
Seasonal utilization rate of manure of N (*MR*)		25–35%	Technical guidelines
Seasonal utilization rate of manure of P (*MR*)		30–35%	
Ratio of manure nutrients to total quantity of fertilizer nutrients of N/P (*MP*)		50%	Zheng, 2019; Zhu, 2020
Annual supply of N per unit pig		11.84 kg	CPSC, 2009
Annual supply of P per unit pig		2.37 kg	
Nutrient retention rate of N (*RR*)		63.50%	Technical guidelines
Nutrient retention rate of P (*RR*)		68.50%	
Proportion of fertilizer nutrients in the total nutrient demand (*FP*)	Wuqiang	45%	
	Shunyi	35%	
	Junan	45%	
	Qingzhou	35%	

The *AMAC* value refers to the cropland area that is required to absorb parts or whole animal manure from livestock and poultry farming (MOA 2018) and was calculated as follows:

2 $$AMAC=\frac{\Sigma \left(BQ\times {Q}_{n}\right)\times RR\times MR}{ND\times FP\times MP}$$

where $AMAC$ is the animal manure absorption capacity, *BQ* refers to total breeding quantity (Table S2), *Q*_*n*_ represents excretion of each livestock and poultry *n* (Table S2), *RR* is nutrient retention rate (Table 4), *ND* is nutrient demand from crops per unit area of land (Table S1), *FP* is the proportion of fertilizer nutrients in the total nutrient demand (Table 2), *MP* is the ratio of manure nutrients to total quantity of fertilizer nutrients, and *MR* is seasonal utilization rate of manure. The annual supply of nitrogen and phosphorus per unit pig (11.84 kg and 2.37 kg, respectively) was calculated based on average value of excretion coefficient (Table 3) of different subspecies of pig reared in northern China, which was multiplied by the breeding days (considered as 365 days).

### Risk warning value

Excessive nitrogen (N) and phosphorus (P) applied to soil will cause nutrient leaching, which can cause environmental pollution. By comparing the actual amount of nutrients provided by livestock manure (*NM*) and the actual nutrients demand of crop from manure (*NCP*), the risk warning value (*R*) was used to evaluate the degree of decoupling between livestock production and crop planting (DNEC and MEP 2002). The *R* value was calculated as follows:

3 $$R=\frac{NM}{NCP}$$

*R* was classified into six grades (DNEC and MEP 2002), as shown in Table 5.

**Table 5:** Classification of the risk warning value (*R*)

*R* value	≤ 0.4	0.4–0.7	0.7–1.0	1.0–1.5	1.5–2.5	> 2.5
Decoupling level	I	II	III	IV	V	VI
Decoupling degree	None	Slight	Little	A bit severe	Severe	Very severe

DNEC and MEP (2002)

## Results

### Comparison of the theoretical *LCC* and actual breeding quantity in four cases

**Fig. 2:**
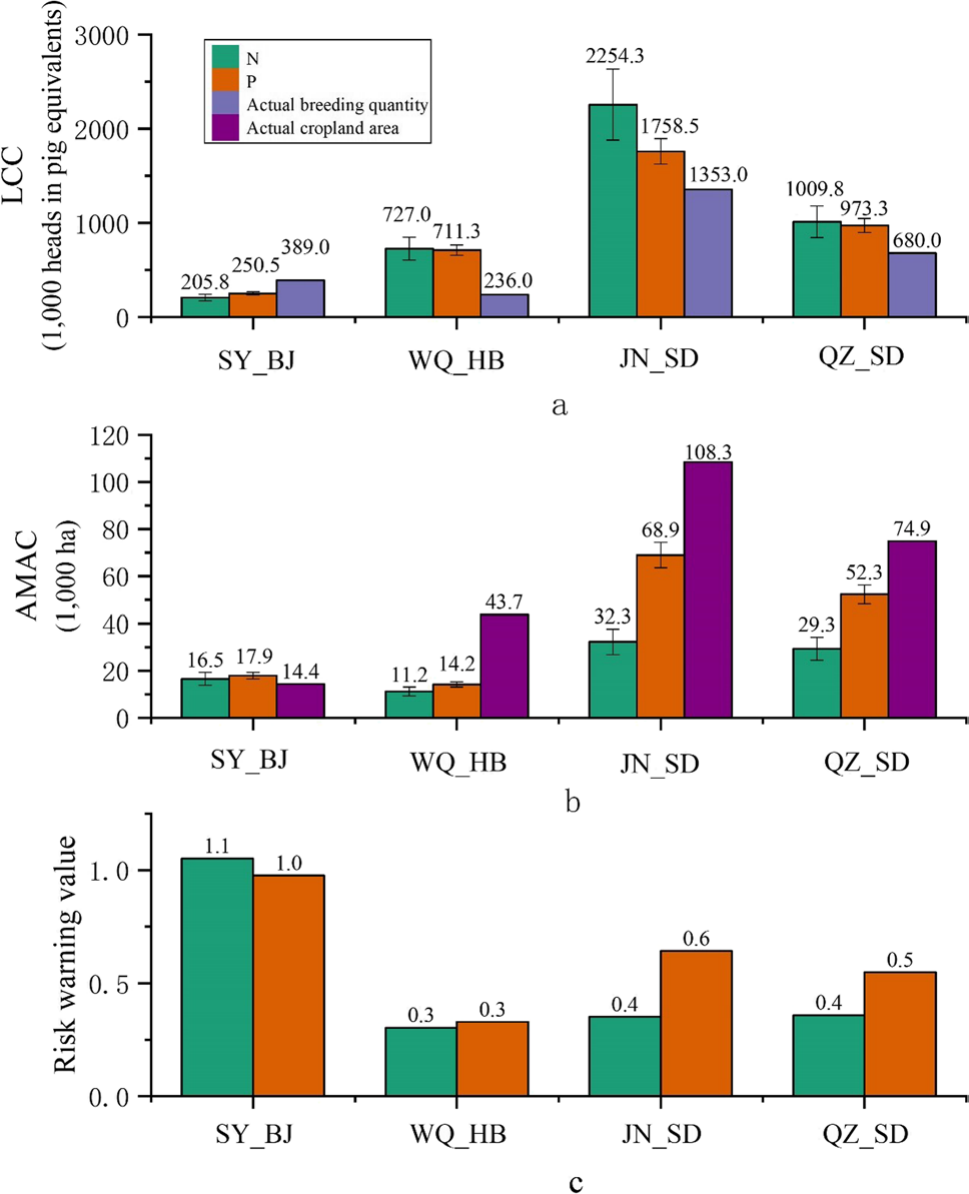
**a** Land carrying capacity (*LCC*), **b** animal manure absorption capacity (*AMAC*), and **c** risk warning value in the four studied sites in the HHH region

Figure 2a shows the theoretical value of *LCC* calculated based on nitrogen and phosphorus demand of crop from manure in the four cases of the HHH region. The *LCC* in SY_BJ was 205.8 ± 33.8 thousand heads in pig equivalents based on nitrogen and 250.5 ± 19.0 thousand heads in pig equivalents based on phosphorus, which was much lower than the actual breeding quantity of 389.0 thousand heads in pig equivalents. The theoretical *LCC* values in the case of SY_BJ was less than 47.1% calculated by nitrogen and 35.6% calculated by phosphorus than the actual breeding quantity. Contrary results were found in the other three cases (JN_SD, QZ_SD, and WQ_HB), where *LCC* values were higher than the local actual breeding quantity. The *LCC* values in WQ_HB were 727.0 ± 121.0 thousand heads in pig equivalents and 711.3 ± 54.8 thousand heads in pig equivalents based on nitrogen and phosphorus, respectively, which were higher than the actual breeding quantity of 236.0 thousand heads in pig equivalents. There was some potential for livestock breeding in this case with 491.0 thousand heads in pig equivalents based on nitrogen and 475.3 thousand heads in pig equivalents based on phosphorus, respectively. The *LCC* values in JN_SD were 2254.3 ± 375.8 thousand heads in pig equivalents based on nitrogen and 1758.5 ± 135.5 thousand heads in pig equivalents based on phosphorus, which were higher than local actual breeding quantity of 1353.0 thousand heads in pig equivalents. There was an increased potential for local livestock breeding with 901.3 thousand and 405.5 thousand heads in pig equivalents based on nitrogen and phosphorus, respectively. In QZ_SD, the *LCC* values were 1009.8 ± 168.3 thousand heads in pig equivalents and 973.3 ± 74.3 thousand heads in pig equivalents calculated by nitrogen and phosphorus, respectively. According to the actual breeding quantity in QZ_SD of 680.0 thousand heads in pig equivalents, there was some potential for local livestock breeding with 329.8 thousand and 293.3 thousand heads in pig equivalents on average based on nitrogen and phosphorus, respectively.

### Comparison of theoretical *AMAC* and actual cropland area in four cases

The *AMAC* results were shown in Fig. 2b. The *AMAC* values for SY_BJ were 16.5 thousand ha and 17.9 thousand ha based on nitrogen and phosphorus, respectively, which were higher than the actual cropland area of 14.4 thousand ha. The result calculated from the actual amount of livestock and poultry was 14.8% larger calculated based on nitrogen and 24.5% larger calculated based on phosphorus than the actual local cropland area. This indicates that livestock and poultry manure had been heavily loaded in this area. Different *AMAC* results were found in the other three cases. In WQ_HB, the *AMAC* values were 11.2 thousand ha based on nitrogen and 14.2 thousand ha based on phosphorus, which were lower than the actual cropland area of 43.7 thousand ha. The *AMAC* values in JN_SD were 32.3 thousand ha calculated by nitrogen and 68.9 thousand ha calculated by phosphorus. Compared with actual local cropland area of 108.3 thousand ha, there was some potential to load the manure excreted by livestock and poultry farming in this area. The *AMAC* values in QZ_SD were 29.3 thousand ha based on nitrogen and 52.3 thousand ha based on phosphorus, which were lower than the actual cropland area of 74.9 thousand ha, with enough cropland area to load the manure excreted from livestock and poultry farming.

### Risk assessment

In order to assess the decoupling of cropland and livestock in each studied case in the HHH region, we drew upon the research results of DNEC (2002) to build a risk warning classification system (see Fig. 2c). Consistent with the results of the carrying capacity, the *R* values in SY_BJ calculated based on nitrogen and phosphorus were both over 1, indicating a bit severe risk of decoupling. The *R* values of the other three cases were all within grade II, and the risk was low. Among them, the results based for the nitrogen and phosphorus in WQ_HB were both 0.3 and categorized as grade I, indicating no decoupling risk in this case. The risk in JN_SD was categorized as grade I with an *R* value of 0.35 based on nitrogen, indicating no risk of decoupling, and the value based on phosphorus was 0.64, which was within grade II, indicating a slight risk of decoupling. The warning values in QZ_SD were 0.36 (grade I) and 0.55 (grade II) based on nitrogen and phosphorus respectively, indicating no risk and slight risk of decoupling, respectively.

## Discussion

### Assessment of carrying capacity based on the different indexes

In this study, we used two carrying capacity indexes (*LCC* and *AMAC*) based on nitrogen and phosphorus and *R* value to assess the decoupling of cropland and livestock at the local scale in the HHH region. The *LCC* value of SY_BJ showed nitrogen as the limiting factor, whereas the other three cases were constrained by phosphorus (Fig. 2a). The difference in the limiting factors in these four cases is due to the nutrients required by the crop, considering both crop types and cropland area. Nitrogen derived from excrement contributes more to the plant nutrient cycle than phosphorus because nitrogen is included within nucleic acids, chlorophyll, urea, and 16% of proteins (Lassaletta et al. 2014; Majee et al. 2019; Wang et al. 2021a, b). Different results were found with the *AMAC* that phosphorus was considered as the limiting factor (except *LCC* of SY_BJ) in four counties (Fig. 2b). This was because of the larger nitrogen content in livestock and poultry manure compared with the phosphorus content (Zhu et al. 2020). Therefore, the assessment of coupling degree of livestock and cropland in four cases should consider both nutrients (nitrogen and phosphorus), which seems more robust than those based on only one nutrient (Peng and Bai 2013).

The *R* value is a further verification of the calculation method of carrying capacity. In this study, nutrient demand of crop and nutrient supply of manure were considered to calculate the *R* value as previous studies by Zheng et al. (2019) and Zhu et al. (2020), and the classification standard was the same as that used by Wang et al. (2017). The *R* results showed that the risk of phosphorus was higher than that of nitrogen, which was mainly due to the larger contribution of nitrogen to the plant nutrient cycle than phosphorus (Lassaletta et al. 2014; Li and Liu (2020)).

### Factors influencing the decoupling of livestock and cropland at the local scale

Different results of coupling at local scale were found in the four cases in the HHH region. This could be explained by some key economic and technical drivers and barriers for coupling of crop and livestock production systems. In this study, the case distributed in peri-urban areas (e.g., SY_BJ) could not couple well with lower *LCC* compared with the other three cases (JN_SD, QZ_SD, and WQ_HB), indicating that urbanization expansion reduces the area of cropland to absorb the animal manure, and urbanization were the main factors for decoupling at local scale. Additionally, higher consumption for animal products was found in high-income regions, which further indicated that economic level influenced the decoupling of livestock and cropland at the local scale (Bai et al. 2022). Urbanization was considered as a threat to cropland availability and food security (Tan et al. 2005; Gardi et al. 2015; Shi et al. 2016; Zhu et al. 2020; Kong et al. 2021). But contrary viewpoint was found that urbanization plays an important role in the new recoupling of livestock and croplands (Jin et al. 2021) through releasing cropland for large-scale farming in China (Wang et al.2021a, b). In the new coupling system caused by urbanization, machinery has been invested for manure transportation and application to local cropland (Yu et al. 2020; Ren et al. 2021). This sounds to reduce transportation costs, which is also a key factor influencing the coupling degree at the county scale (Hou et al. 2021), where livestock is often located near urban areas to satisfy the demand of human diets, while cropland is located in rural areas. The long-distance transport cost may be greater than the value of manure, so the livestock manure is usually recommended to be used as fertilizer in the nearby farmland. The farthest transport distance for manure fully adsorbed in cropland is within 2 km, which is considered as a reasonable distance to couple the livestock and cropland.

Recoupling the livestock and cropland could reduce the risk of high nutrient losses from Chinese livestock system, and technical interventions are needed to increase resource-use efficiency and lessen negative environmental impacts. There are a number of available technologies that would allow recovery of nutrients and energy from manure using the infrastructure of farms. The common treatment technologies are composting and anaerobic fermentation. Due to low capital investment and easy operation, composting does not need a complicated and high-tech infrastructure so could be regarded as the first choice in many countries. However, under conventional composting methods considerable amount of nitrogen can be lost through leaching and ammonia volatilization and would cause GHG emissions. Anaerobic digestion has long been introduced as an environmentally friendly option for manure management for mitigating manure’s impacts, producing bioenergy, and generating digestate as a biofertilizer. Moreover, biogas plants can decrease the average distance between plants and farms so as to minimize negative environmental impacts.

### Uncertainties and prospects

In this study, we applied several local coefficients, such as the soil nutrient grade and the ratio of manure nutrients to total quantity of fertilizer nutrients (*MP*), to make the results more accurate. Furthermore, data relating to planting (Table S1), and livestock and poultry breeding (Table S2) were collected from the local statistical yearbooks. The coefficients of *MP* and *FP* were based on the studies in the HHH region (Tables 4 and 5). However, there were also considerable uncertainties in the estimation of the carrying capacities in this study. For instance, the excretion coefficients (Table 3) and the coefficients of *MR* and *RR* (Table 4) were available only for the national scale, not for the local situation, but the *LCC* and *AMAC* results are highly context specific. Thus, the coefficients may not be sufficient to estimate the local carrying capacities from the complex and diverse farmland in the HHH region. The results of carrying capacities may be under- or over-estimated with the direct use of different coefficients. Despite these uncertainties, the above analysis identified the decoupling case, based on the combined use of various indicators. The optimization methods will be established to explore the recoupling options of the decoupled case at the local scale.

## Optimization

### Optimization methods

In view of the discrepancy between the *LCC* or the *AMAC* and local actual agricultural conditions, some options (e.g., the ratio of manure to fertilization and the breeding quantity) were optimized to recouple the livestock and croplands. Subsequently, the optimal *LCC* or *AMAC* values were assessed according to *R*.

#### Adjust the ratio of manure to fertilization

To recouple the livestock and cropland, the *LCC* value from Eq. (1) should be higher than or equal to the actual local livestock and poultry farming amount. One recoupling option is to increase the *MP* of N and P to greater than 50%. The first step for this optimization was to set the data for parameters in Eq. (1). Then Eq. (1) was rewritten to Eq. (4) for *LCC*(N) and Eq. (5) for *LCC*(P), respectively.

4 $$LCC\left(\mathrm{N}\right)=40.8\times MP$$

5 $$LCC\left(\mathrm{P}\right)=47.3\times MP$$

To recouple the livestock and croplands, the *AMAC* value from Eq. (2) should be lower than or equal to the actual local planting area. One option was to increase the *MP* of N and P, which should be higher than 50%. The first step for this optimization was to set the data for parameters in Eq. (2). Then Eq. (2) was rewritten as Eq. (6) for *AMAC*(N) and Eq. (7) for *AMAC*(P), respectively.

6 $$AMAC\left(\mathrm{N}\right)=8.1\times {MP}^{-1}$$

7 $$AMAC\left(\mathrm{P}\right)=9.4\times {MP}^{-1}$$

#### Adjust the local breeding amount

To recouple the cropland and livestock, the actual breeding amount should be reduced to be lower than or equal to the theoretical result of *LCC*. This means that the reduced amount of local stock (*RS*, shown in Eq. 8) should be higher than or equal to the targeted amount of pig equivalent to be reduced (*C*, shown in Eq. 8), which is determined by comparing the theoretical *LCC* with the actual pig equivalent in decoupled regions. The reduced amount of the total local stock (*RS*) was calculated by decreasing the quantity of various livestock and poultry (*X*). The appropriate reduction rate of livestock and poultry breeding stock was calculated as follows:

8 $$\begin{array}{c}RS\geq C\\Y=\sum\left(\frac{a\;\times\;X}b\right)\end{array}$$

where *RS* is the reduced amount of pig equivalents, *C* represents the targeted amount of pig equivalents to be reduced, *X* is the reduction rate of livestock and poultry breeding stock, *a* is the actual breeding quantity of local livestock and poultry, and *b* denotes the conversion ratio of pig equivalent.

### Optimizing the decoupling cases

#### Adjusting the ratio of manure to total fertilization

**Fig. 3:**
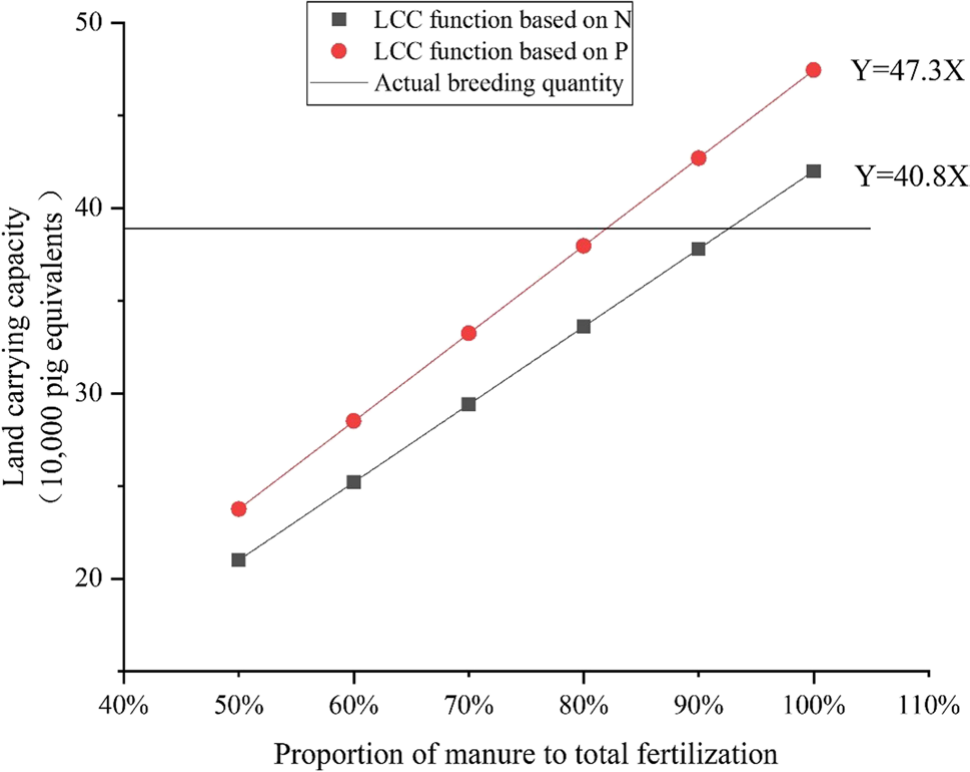
Optimization of the land carrying capacity (*LCC*)

The decoupling of livestock and cropland at the local scale was found in the case of SY_BJ, which could be optimized by adjusting the proportion of manure to fertilization based on Eqs. (4) and (5) (Fig. 3). Compared with the actual breeding quantity of 389.0 thousand heads in pig equivalents, the proportion of manure to fertilization calculated by nitrogen should be larger than 95.34% of manure from local livestock and poultry, which should be returned to local cropland. The proportion of manure in fertilization calculated by phosphorus should be larger than or equal to 81.97% to match the actual local breeding quantity.

**Fig. 4:**
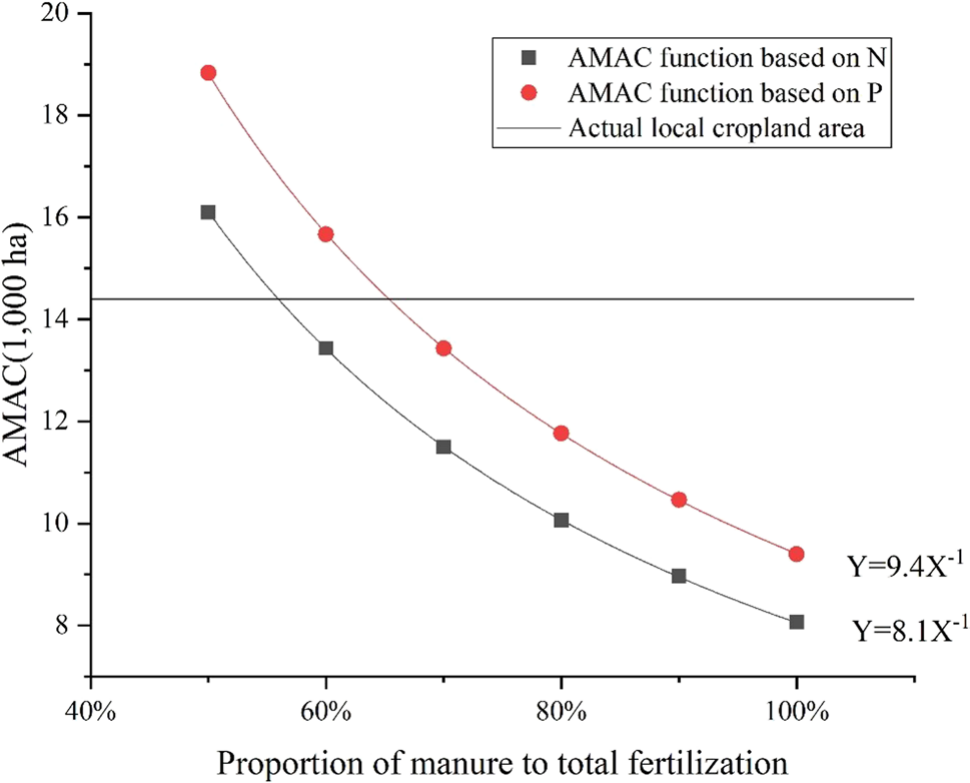
Optimization of the animal manure absorption capacity (*AMAC*)

The technology of composting was used to handle animal manure in the case of SY_BJ. The nutrient retention rates of nitrogen and phosphorus in the composting technology were considered to be 62% and 32.5%, respectively (MOA 2018). The actual local cropland area of 14,400 ha was regarded as the upper limit based on Eqs. (6) and (7) (Fig. 4). Based on nitrogen, the proportion of manure in fertilization should be greater than or equal to 55.91% to reduce the *AMAC* below the actual local planting area. Based on phosphorus, *AMAC* should be larger than or equal to 65.33% to match the actual planting area.

#### Adjust the local breeding quantity

A comparison of *LCC* results in SY_BJ (205.8 thousand heads in pig equivalent and 250.5 thousand heads in pig equivalents based on nitrogen and phosphorus, respectively) with the actual breeding quantity (389 thousand heads in pig equivalent), the potential to reduce the breeding quantity based on nitrogen (179,000 heads in pig equivalents) was larger than that calculated based on phosphorus (152,000 heads in pig equivalents). Correspondingly, we put the larger reduced amount of 179,000 heads in pig equivalents calculated by nitrogen into Eq. (8). The appropriate reduction rate of livestock and poultry breeding was then calculated, considering the overall reduction rate of livestock and poultry breeding as an assumption in this study. The quantity of livestock and poultry breeding in SY_BJ needs to be reduced by at least 46% to recouple the livestock and cropland. With the optimized quantity of livestock and poultry, the *R* value in the case of SY_BJ could be reduced to 0.48 and 0.45 based on nitrogen and phosphorus, respectively. These results could be categorized as risk grade II, which was lower than the original *R* value (above 1). In this case, the risk of decoupling would be decreased from a bit severe to slight.

### Optimization and implications for manure management

The carrying capacity indexes and the *R* value could be used to identify cases in which livestock and cropland are coupled or decoupled. The coupling degree in SY_BJ was lower than the other three cases based on the lower theoretical *LCC* and higher *AMAC*. This result was similar to the findings by Zheng et al. (2019) that the livestock industry in Beijing area needed to be strictly controlled because of the grade IV or V classifications. Therefore, the nutrient demand of crop for growth and the nutrient availability of livestock manure in the case of SY_BJ should be scientifically calculated in order to reduce the environmental risks associated with livestock farming.

Two optimization options in this study were put forward to decrease pressure of excessive manure on the agricultural environment in the case of SY_BJ. The first option is to match the theoretical carrying capacities with the actual cropland area or breeding quantity by adjusting the ratio of manure to fertilization. The optimal ratio for mixing organic and chemical fertilizers can improve the utilization of excrement and realize zero growth of chemical pollution (Wang et al. 2021a, b; Yang et al. 2021a, b). A higher ratio of manure to fertilization (95.34%) was calculated by *LCC* based on nitrogen compared with a ratio of manure to fertilization (65.33%) by the *AMAC* based on phosphorus. Comparison of these two calculation equations reveals that the ratio of manure to fertilization after the optimization of *AMAC* was less than that of the *LCC*, mainly because the *AMAC* result was calculated by the average nutrient requirement per unit area of land, which may ignore some high-nutrient-demand crops. Returning excrement to farmland can supplement, but not completely replace, chemical fertilizers with regard to the need for nutrients (Wang et al. 2021a, b). This source of nutrients is used effectively for crop growth and represents a great opportunity to reduce the purchase of (and requirement for) chemical fertilizers and potentially improve farmer incomes (Jia et al. 2015). In addition, manure can promote the microbial fixation of bioavailable N and improve the soil micro-environment (Zhang et al. 2016). A reasonable rate of livestock manure returning to the cropland can help construct an integrated planting–breeding system to increase livestock breeding potential (Zheng et al. 2019).

The second option is adjusting the actual breeding quantity to recouple the livestock and cropland in the case of SY_BJ. The phosphorus content in livestock manure is generally lower than that of nitrogen, so the *AMAC* result calculated by P was used for optimization. The optimization equation was constructed according to the theoretical *LCC* by reducing the breeding quantity of different types of livestock and poultry. An appropriate rate to reduce breeding quantity (at least 46%) was suggested to recouple the cropland and livestock in the case of SY_BJ. The decrease of *R* value means that the adjusting the breeding quantity of livestock is an effective way to realize the recoupling in this case. Reducing at least 46% of breeding quantity could be proposed in Beijing as a similar strategy has been proposed in the European Union that halving meat consumption for human healthy diet (Gu 2022).

## Conclusions

This study estimated the decoupling between livestock and cropland systems at the county scale, as well as the optimal options for future recoupling in the identified decoupled case. Based on the index of *LCC*, *AMAC*, and the *R* value, the case of SY_BJ was decoupled while the cases of WQ_HB, JN_SD, and QZ_SD were coupled at the local scale. To recouple the livestock and cropland in the case of SY_BJ, the nitrogen and phosphorus surplus from livestock sectors need to be lowered. Some optimal options have been suggested to increase nutrient use efficiency in the livestock–crop system by adjusting the ratio of manure to total fertilization and the local breeding quantity. Different ratios of manure to fertilization were found by *LCC* based on nitrogen (95.34%) and by the *AMAC* based on phosphorus (65.33%). The *R* value in the case of SY_BJ could be categorized as risk grade II, decreasing from a bit severe to slight through more than 46% of breeding quantity of livestock and poultry. Further analysis is needed to identify how measures or policies can best reduce nutrient surpluses and recouple cropland and livestock at the local scale, close to urban area.

## Supplementary Information

Below is the link to the electronic supplementary material.

Supplementary file1 (DOCX 28.8 KB)

## Data Availability

Please contact the authors for data requests.
