# Supplementary material for: Evaluation of the local decoupling of livestock and cropland in the Huang-Huai-Hai region
Source: Environ Sci Pollut Res Int. 2022 Jul 25;29(60):90911–22. doi: 10.1007/s11356-022-21993-2 (PMC9722824; doi:10.1007/s11356-022-21993-2)
Supplement: Supplementary file 1 — Supplementary file1 (DOCX 28.8 KB) [file 11356_2022_21993_MOESM1_ESM.docx]

Table S1. Crop agriculture statistics in the study area

| County | Crop species | | Total crop yield (*Y*) (Ton) | Planting area  (ha) | Plant nutrient requirement /N (Ton) (*ND*) | Plant nutrient requirement /P (Ton) (*ND*) |
| --- | --- | --- | --- | --- | --- | --- |
|  |  |  |  |  |  |  |
| Wuqiang | Staple crops | Wheat | 105654.50 | 16523.30 | 3169.60 | 1056.50 |
|  |  | Corn | 133385.60 | 23173.70 | 3067.90 | 400.20 |
|  |  | Millet | 314.40 | 314.00 | 11.90 | 1.40 |
|  |  | Soybeans | 560.10 | 210.00 | 40.30 | 4.20 |
|  |  | Cotton | 107.00 | 106.00 | 12.50 | 3.30 |
|  |  | Potato | 204.10 | 7.60 | 1.00 | 0.20 |
|  |  | Sorghum | 0.02 | 0.10 | 93.10 | 29.90 |
|  | Vegetables | Cucumber | 33255.00 | 395.00 | 35.50 | 10.80 |
|  |  | Tomato | 10763.00 | 182.00 | 46.20 | 9.70 |
|  |  | Green Pepper | 9058.00 | 165.00 | 39.00 | 11.50 |
|  |  | Eggplant | 11472.00 | 216.00 | 14.50 | 3.00 |
|  |  | Radish | 5186.00 | 77.00 | 6.30 | 1.20 |
|  |  | Green Chinese onions | 3323.00 | 83.00 | 42.90 | 7.60 |
|  |  | Green beans | 1.50 | 0.60 | 1.10 | 0.20 |
|  |  | Sweet potato | 1858.10 | 48.80 | 1.90 | 1.30 |
|  |  | Celery | 1341.00 | 44.00 | 1.20 | 0.30 |
|  |  | Rape | 1828.00 | 57.00 | 0.10 | 0.04 |
|  |  | Spinach | 4637.00 | 148.00 | 320.50 | 39.50 |
|  |  | Chinese cabbage | 24889.00 | 341.00 | 0.0005 | 0.0003 |
|  |  | Cabbage | 1339.00 | 22.00 | 0.10 | 0.01 |
|  |  | Pumpkin | 2194.00 | 24.00 | 6.50 | 3.30 |
|  |  | Wax gourd | 134.00 | 2.00 | 5.40 | 1.90 |
|  |  | Garlic | 5226.00 | 82.00 | 4.90 | 0.50 |
|  | Fruits | Peach | 524.00 | 29.00 | 16.70 | 8.30 |
|  |  | Grape | 256.00 | 34.00 | 37.30 | 17.40 |
|  |  | Apple | 393.00 | 52.00 | 5.50 | 0.70 |
|  |  | Pear | 21.00 | 7.00 | 98.70 | 39.50 |
|  | Economic crops | Oil plants | 4458.00 | 1378.00 | 0.20 | 0.10 |
| Shunyi | Staple crops | Wheat | 21483.70 | 4078.50 | 644.50 | 214.80 |
|  |  | Corn | 31906.60 | 5056.10 | 733.90 | 95.70 |
|  | Vegetables | Cucumber | 30980.00 | 459.20 | 86.70 | 27.90 |
|  |  | Tomato | 79564.00 | 964.90 | 262.60 | 79.60 |
|  |  | Green pepper | 5137.20 | 85.70 | 26.20 | 5.50 |
|  |  | Eggplant | 30010.00 | 444.90 | 102.00 | 30.00 |
|  |  | Chinese cabbage | 47382.00 | 526.70 | 71.10 | 33.20 |
|  |  | Radish | 5513.68 | 91.90 | 15.40 | 3.10 |
|  |  | Green Chinese onions | 7182.80 | 119.80 | 13.60 | 2.60 |
|  |  | Garlic | 609.00 | 33.90 | 5.00 | 0.90 |
|  | Fruits | Watermelon | 60064.80 | 834.70 | 480.50 | 198.20 |
|  |  | Peach | 6271.70 | 294.90 | 13.20 | 2.10 |
|  |  | Grape | 3461.20 | 177.40 | 25.60 | 17.70 |
|  |  | Apple | 7418.70 | 707.70 | 22.30 | 5.90 |
|  |  | Pear | 12746.80 | 524.70 | 59.90 | 29.30 |
|  | Economic crop | Oil plant | 60.40 | 20.70 | 4.30 | 0.50 |
| Junan | Staple crops | Wheat | 185106.00 | 33170.60 | 5553.20 | 1851.10 |
|  |  | Rice | 1434.00 | 180.20 | 31.50 | 11.50 |
|  |  | Corn | 236114.00 | 34150.60 | 5430.60 | 708.30 |
|  |  | Millet | 325.00 | 63.70 | 12.40 | 1.40 |
|  |  | Soybeans | 5424.00 | 1963.00 | 390.50 | 40.60 |
|  |  | Cotton | 177.00 | 145.50 | 20.70 | 5.40 |
|  |  | Barley | 181.00 | 36.60 | 5.40 | 1.80 |
|  |  | Potato | 44529.00 | 5421.80 | 222.60 | 39.20 |
|  | Vegetables | | 259745.00 | 5192.10 | 941.60 | 229.20 |
|  | Fruits | Peach | 21441.00 | 978.70 | 45.00 | 7.10 |
|  |  | Grape | 6864.00 | 165.70 | 50.80 | 35.10 |
|  |  | Apple | 16414.00 | 646.80 | 49.20 | 13.10 |
|  |  | Pear | 3618.00 | 116.30 | 17.00 | 8.30 |
|  | Economic crops | Oil plant | 126050.00 | 25753.50 | 9063.00 | 1118.10 |
|  |  | Tobacco | 1035.00 | 405.10 | 39.80 | 5.50 |
|  |  | Tea | 1266.00 | – | 81.00 | 11.10 |
| Qingzhou | Staple crops | Wheat | 80000.00 | 14047.00 | 2400.00 | 800.00 |
|  |  | Corn | 141159 | 22209.00 | 3246.70 | 423.50 |
|  |  | Millet | 2626.00 | 806.00 | 99.80 | 11.60 |
|  |  | Soybeans | 1817.00 | 788.00 | 130.80 | 13.60 |
|  |  | Cotton | 47.00 | 47.00 | 5.50 | 1.40 |
|  |  | Sorghum | 91.00 | 34.00 | 2.40 | 1.20 |
|  |  | Potato | 3308.00 | 563.00 | 16.50 | 2.90 |
|  | Vegetables | | 1796724.00 | 31816.00 | 6513.10 | 1585.60 |
|  | Fruits | Peach | 33255.00 | 2373.00 | 69.80 | 11.00 |
|  |  | Grape | 489.00 | 71.00 | 3.60 | 2.50 |
|  |  | Apple | 6329.00 | 258.00 | 19.00 | 5.10 |
|  |  | Persimmon | 15766.00 | 1775.00 | 126.10 | 47.30 |
|  |  | Pear | 1100.00 | 67.00 | 5.20 | 2.50 |
|  | Economic crop | Oil plant | 68.00 | 32.00 | 4.90 | 0.60 |

Data source: Statistical yearbook of Beijing Shunyi, Shandong Linyi, Weifang, Hebei Hengshui; Plant nutrient requirements were calculated according to Table 1.

Table S2. Statistics of livestock and poultry breeding in the study area

| County | Livestock species | Total breeding quantity (100 heads) (*BQ*) | Nitrogen excretion (Ton) (*Q_n_*) | Phosphorus excretion (Ton) (*Q_n_*) |
| --- | --- | --- | --- | --- |
| Wuqiang | Pig | 528.00 | 238.13 | 89.76 |
|  | Cow | 213.00 | 1301.43 | 214.49 |
|  | Cattle |  |  |  |
|  | Sheep | 297.00 | 67.72 | 13.37 |
|  | Poultry | 19613.00 | 539.36 | 225.55 |
| Shunyi | Pig | 2537.00 | 1144.00 | 431.20 |
|  | Cow | 60.00 | 1270.80 | 209.40 |
|  | Cattle | 148.00 |  |  |
|  | Sheep | 610.00 | 139.00 | 27.40 |
|  | Laying hen | 4083.00 | 146.40 | 61.20 |
|  | Broiler chicken | 125.00 |  |  |
|  | Duck | 11170 |  |  |
| Junan | Pig | 1017.00 | 4587.10 | 1729.10 |
|  | Cow | 0.60 | 1423.60 | 234.60 |
|  | Cattle | 22.70 |  |  |
|  | Sheep | 104.00 | 236.90 | 46.80 |
|  | poultry | 5367.00 | 1475.90 | 617.20 |
| Qingzhou | Pig | 218.60 | 985.90 | 371.60 |
|  | Cow | 13.80 | 843.20 | 139.00 |
|  | Cattle |  |  |  |
|  | Sheep | 98.50 | 224.60 | 44.30 |
|  | Poultry | 9045.00 | 2487.30 | 1040.20 |

Data source: Statistical yearbook of Beijing Shunyi, Shandong Linyi, Weifang, Hebei Hengshui; Nitrogen and phosphorus excretions are calculated according to Table 3.
